# Prevalence, etiology, diagnosis, treatment and complications of supernumerary teeth

**DOI:** 10.4317/jced.51499

**Published:** 2014-10-01

**Authors:** Fadi Ata-Ali, Javier Ata-Ali, David Peñarrocha-Oltra, Miguel Peñarrocha-Diago

**Affiliations:** 1 DDS, MS. Valencia University Medical and Dental School; 2 DDS, MS, MPH, PhD. Public Dental Health Service. Arnau de Vilanova Hospital. Master in Oral Surgery and Medicine. Master in Oral Surgery and Implantology. Collaborating professor of the Master in Oral Surgery and Implantology. Valencia University Medical and Dental School; 3 DDS, MS, PhD. Master in Oral Surgery and Implantology. Collaborating professor of the Master in Oral Surgery and Implantology. Valencia University Medical and Dental School; 4 DDS, PhD, MD. Professor, Director of the Master of Oral Surgery and Implantology. Valencia University Medical and Dental School, Valencia, Spain

## Abstract

The aim of this article was to review the literature on supernumerary teeth, analyzing their prevalence, etiology, diagnosis, treatment and possible complications. An electronic search was made in the Pubmed-Medline database up to January 2014 using the key search terms “multiple supernumerary teeth” (n=279), “prevalence supernumerary teeth” (n=361), and “supernumerary teeth” (n=2412). In addition to the articles initially identified, others were included in the review proceeding from a manual search and from any references considered of relevance. Supernumerary teeth are those that exceed the normal dental formula. They are more common in men, more common in the upper maxilla, and more prevalent in permanent dentition. Complications associated with supernumerary teeth include dental impaction, delayed eruption, ectopic eruption, overcrowding, spacing anomalies and the formation of follicular cysts. The treatment of supernumerary teeth depends on their type, position, and possible complications, detected clinically and radiographically. No clear consensus exists as to the best time to extract unerupted supernumerary teeth.

** Key words:**Hyperdoncia, supernumerary teeth, impacted teeth, treatment, permanent teeth, deciduous teeth.

## Introduction

Supernumerary teeth are those that exceed the normal dental formula. This phenomenon is also known as hyperdoncia and can occur in solitary or multiple form, may be unilateral or bilateral, and affect one or both maxillas (1). These teeth are more prevalent among men than women in a proportion of 2:1. The prevalence of supernumerary teeth is 0.3-0.8% in deciduous dentition and 1.5-3.5% in permanent dentition (1,2). Rao and Chidzonga (3) state that the etiology of supernumerary teeth is multifactorial, a combination of environmental and genetic factors.

Complications associated with supernumerary teeth include impaction, delayed eruption, ectopic eruption, overcrowding, spacing anomalies and the formation of follicular cysts (4,5). Some cases of supernumerary teeth are asymptomatic and detected casually in the course of radiographic examination (6). Both clinical and radiographic examination is essential for detecting supernumerary teeth although recently computerized tomography has been used as a complimentary diagnostic test. Treatment depends on their type, position and possible complications, identified both clinically and radiographically. Although surgical extraction is the most common treatment, another option is to reposition supernumerary teeth in the dental arch.

The aim of this article was to review the literature on supernumerary teeth, analyzing their prevalence, etiology, diagnosis, treatment and possible complications.

## Material and Methods

An electronic search was made in the Pubmed-Medline database until January 2014 using the key search terms “multiple supernumerary teeth” [n=279], “prevalence supernumerary teeth” [n=361] and “supernumerary teeth” [n=2412]. Of the articles initially located for review, others were added proceeding from a manual search and from any references in the review articles thought to be relevant. The titles and abstracts of the articles were reviewed to determine which were relevant and the full text of relevant articles were read thoroughly and subjected to analysis. Studies of patients with supernumerary teeth published during the last 13 years with patient series of more than 20 were included in the review ( Table 1).

**Table 1:**
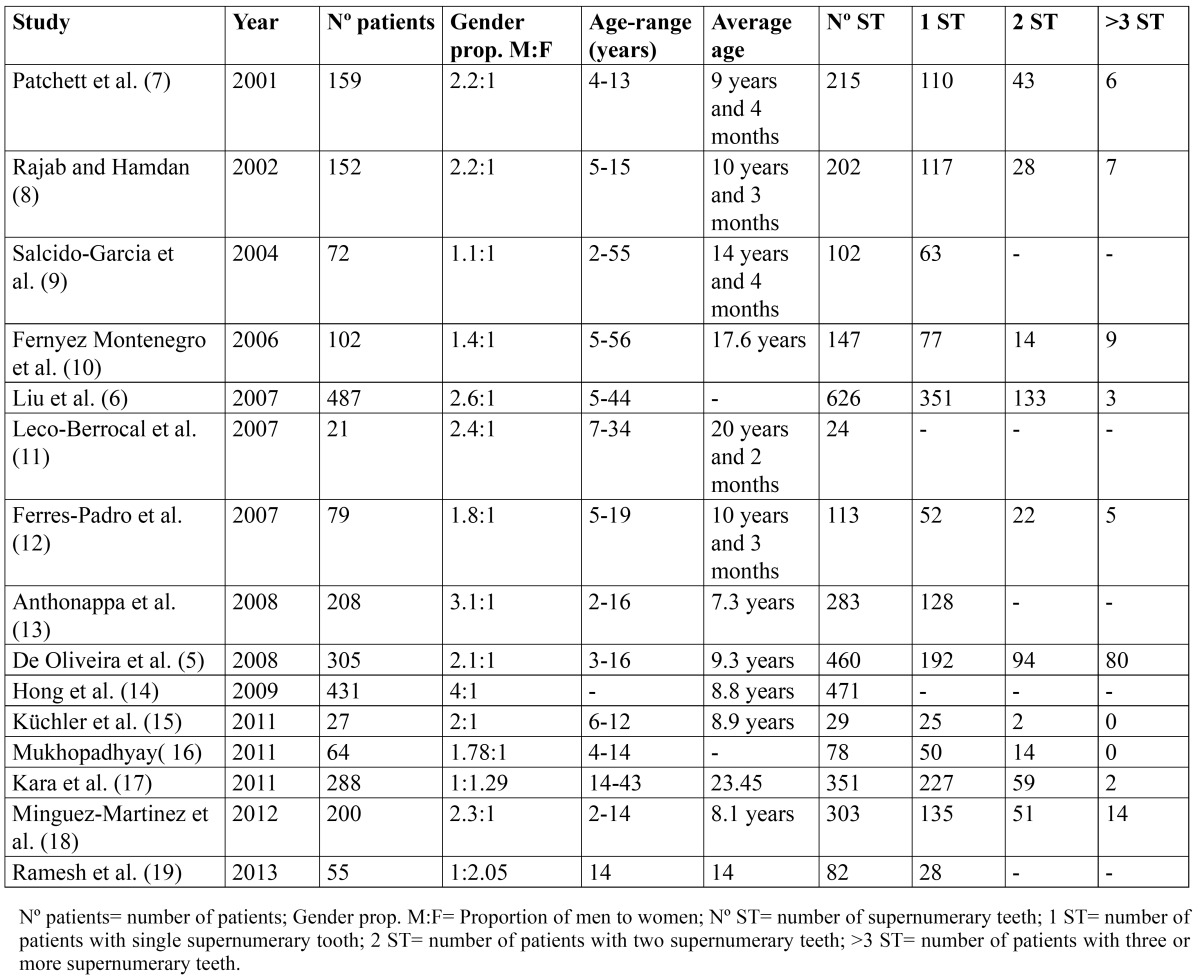
Review of recent studies of patients with supernumerary teeth.

## Prevalence

The prevalence of supernumerary teeth in deciduous dentition is 0.3-0.8%, and in permanent dentition 1.5-3.5% (1,2,14). A meta-analytic study (20) has shown that the value obtained for prevalence depends directly on the diagnostic tools used, panoramic radiography being the most effective diagnostic method. Supernumerary teeth are more prevalent among men than women in a proportion of 2:1 (1,2,6,14,16,21,22), although some authors report this proportion to range from 1.7:1 to 3.1:1 (4,6,8,16,22). The upper maxilla presents supernumerary teeth more frequently than the mandible. A study by Liu *et al*. (6) of 487 patients and 626 supernumerary teeth, found that they were located in the upper maxilla in 92% of the patients. In another study (22) of 283 supernumerary teeth, they were situated in the upper maxilla in 95% of cases, while Mahabob* et al*. (2) analyzed 2,216 patients with 27 supernumerary teeth, 67% of which were situated in the upper maxilla. Minguez-Martínez *et al*. (18) studied 303 supernumerary teeth in 200 patients observing that 88% were situated in the upper maxilla. However, another study of 393 supernumerary teeth found almost the same frequency in the two jaws (23).

## Etiology

The etiology of supernumerary teeth remains unclear and various theories have been postulated to explain how and why they develop. Various studies (5,8,24) have claimed that they are the result of hyperactivity of the dental lamina where the epithelial cells that form supernumerary teeth remain for long periods. Other studies (3,24) show that the main etiological factor is genetic predisposition, having to do with a disorder associated with a dominant autosomal gene. Elsewhere, they are thought to be associated with diverse syndromes or due to phylogenetic theory, environmental factors or tooth germ dichotomy (3,24).

Phylogenetic theory, although it has been discounted as it would only explain single anomalies of ectopic teeth (8), proposes that the presence of supernumerary teeth involves a regression towards now extinct ancestral tissues. While in the process of evolution dentition has passed from polyodonty to oligodonty, dental morphology has become more complex, evolving from homodonty to heterodonty (25). With regard to environmental factors, in tooth germ dichotomy, an imbalance between molecules can cause the tooth germ to divide into two parts, of equal or different size, which will result in either two teeth of the same size or one normal and one dysmorphic tooth (26).

## Complications

Supernumerary teeth can be asymptomatic and only diagnosed casually in the course of radiographic examination (6,8). However, the majority are associated with complications that include dental impaction, delayed eruption [particularly those of tuberculate morphology, located palatally to the upper central incisors] or ectopic eruption of an adjacent tooth, overcrowding [mainly caused by supplementary supernumerary teeth in the anterior region of the upper maxilla], spacing anomalies [for example, a diastema may develop when the supernumerary tooth is situated in the midline of the upper maxilla], ectopic eruption [for example, in the floor of the nasal cavity], dilaceration or abnormal development of the root, or the formation of follicular cysts (4,5,8). Delayed eruption of permanent teeth is the most common complication, while overcrowding, diastema, or root dilaceration are less frequent (27). However, a study (28) that examined 4,133 children by radiography found that the most frequent complication was midline diastema [28.6%].

Seddon *et al*. (27) observed that in 26-52% of cases supernumerary teeth caused delayed eruption of the permanent teeth, while displacement and rotation of adjacent teeth were observed in 28-63% of cases. Another study (5) found that 88.5% of supernumerary teeth involved complications, the most frequent being dental displacement [55.7%], followed by delayed eruption [50.8%], diastemas [21%], tooth rotations [18.7%], retention of deciduous teeth [7.9%] and root resorption [0.3%]. One of the main clinical implications provoked by these teeth is their tendency to interfere with normal occlusal development, as corroborated in several studies (16,17,29), in which retention was produced in 81.1%, 78.8% and 53.8% of cases, respectively.

## Diagnosis

It has been suggested that the sooner supernumerary teeth are diagnosed the better their prognosis will be. The presence of asymmetry indicates the possible presence of a mesiodens that could provoke the retention of the deciduous upper incisors or the ectopic eruption of one or both permanent upper incisors (8). Adequate clinical and radiography examination is crucial to detect supernumerary teeth and computerized tomography has been introduced recently as a complementary diagnostic method. One study used cone beam computerized tomography (CBCT) to examine 487 patients and 626 supernumerary teeth, finding this radiography technique to be very precise as it determined the location of each supernumerary tooth exactly (6). Occlusal or periapical radiography is important for diagnosing supernumerary teeth in the incisor region. The parallelism technique allows detection of the supernumerary tooth position in vestibulo- lingual direction (8).

## Number, Location and Position

Most frequently, patients present a single supernumerary tooth. Various studies (8,16,19,28,29) confirm this fact and have obtained percentages of 89.7%, 77%, 78.1%, 76.8% and 50.90%, respectively. According to Küchler *et al*. (15) cases of a single supernumerary tooth can reach 92.5%. The appearance of two supernumerary teeth in a patient is the second most common occurrence, observed in 7.5% (15), 18.4% (8), 21.9% (16), or 23.1% of cases (29). Multiple supernumerary teeth are rare representing less than 1% of all cases (30). Nevertheless, various studies (19,24) have found multiple supernumerary teeth unassociated with any systemic disorder or syndrome. One study of 283 supernumerary teeth (22), found that 48% of them were in an inverted position, a similar finding to Gündüz *et al*. [37.6%] (29). However, Liu *et al*. (6) and Rajab and Hamdan (8) state that most supernumerary teeth showed normal orientation. Another study (8) observed that the area where supernumerary most commonly appear is the anterior region [89.6%], a similar finding to Mahabob *et al*. (2) [85.7%]; in the canine and premolar regions, the presence of supernumerary teeth was 9% and in the molar area 0.5% (8).

In the horizontal plane, Rajab and Hamdan (8) found that 82.5% of teeth were observed in the palatal/lingual area, a similar finding to Oliveira *et al*. [84.1%] (5). Supernumerary teeth are found in mixed positions in 13.9% of cases and in vestibular position in 1% (8). Another study (13) observed palatal eruption in 79.1% and vestibular in 2.1%.

## Classification

Supernumerary teeth can be classified according chronology, location, morphology and orientation. Garvey *et al*. (1) classify them as single or multiple. Single supernumerary teeth are classed on the basis of their morphology as conical, tuberculate, supplementary and odontomas, the latter being composite or complex. Primosch (31) classified supernumerary teeth as two types according to shape: supplementary or rudimentary; supplementary or eumorphic are those that have a normal shape and size, rudimentary or dysmorphic have an abnormal shape and smaller size and maybe conical, tuberculate or molariform.

According to the location of supernumerary teeth, they can be classified as mesiodens [situated at the midline], paramolar [situated vestibularly between the second and third molars, and distomolar [situated distally of the third molar]. They may show vertical, inverted, or transversal orientations (8).

The most common supernumerary teeth present conical morphology, most usually situated between the upper central incisors (8). One study (5) observed that the supernumerary tooth of conical morphology was the most common [44.5%], followed by tuberculate [38.7%] and supplementary [16.7%]. These data concur with results published by Kara *et al*. (17) [47.3%, 39.9% and 12.8%, respectively] and by Ramesh *et al*. (19) [79.74%, 9.75% and 7.31%, respectively]. However, other studies (8,32) have obtained prevalence data that vary from 31% to 75% for conical, 12-28% for tuberculate, and 4-33% for supplementary teeth.

## Treatment

Opinions vary widely from author to author as to how to treat supernumerary teeth, particularly with regard to the right time for extraction (33). The usual treatment is to extract the supernumerary tooth, although repositioning it in the dental arch may be an alternative option (6,34). Extraction must be performed with care, avoiding any damage to blood vessels and nerves or to anatomical structures such as the maxillary sinus, pterygomaxillary space, or the orbit [and possible fracture] of the maxillary tuberosity. To date there is no clear consensus as to the best time for surgical extraction of an unerupted supernumerary tooth. When a supernumerary tooth is located in the upper anterior zone, surgery is recommended at the age of eight to ten years, when incisor root development is complete (8). Rao and Chidzonga (3) claim that extraction should proceed only when the roots of adjacent teeth are fully developed. Omer *et al*. (35) carried out a study to identify the different changes and complications that can be produced in teeth adjacent to supernumerary teeth, in relation to the degree of root development at the moment of extraction of the supernumerary tooth, in order to determine the best time for extraction. They concluded that the therapeutic option that provokes the fewest complications is the surgical extirpation of unerupted supernumerary teeth when the permanent teeth are in formation stage C according to Demirijian. Another therapeutic option is to keep the supernumerary tooth under observation as long as it does not provoke any complication and does not interfere with function or aesthetics (34).

## Syndromes Associated with Supernumerary teeth

It is rare for hyperdoncia to occur in isolation; it is usually associated with some other disorder such as harelip, cleft palate, or syndromes such as Gardner syndrome, Down syndrome, cleidocranial dysplasia, Zimerman-Laby syndrome or Noonan syndrome (36). A study (37) of 205 patients with harelip and cleft palate found a frequency of supernumerary teeth of 11.7%. Several other studies (38,39) have observed cases of cleidocraneal dysplasia with the presence of supernumerary teeth. The gene responsible for cleidocraneal dysplasia is the RUNX2 and mutations of this gene could explain the existing correlation between the syndrome and the presence of supernumerary teeth (40).

## Conclusions

Supernumerary teeth are more frequent among men than women, more frequent in the upper maxillary, and more prevalent in permanent dentition. Complications associated with supernumerary teeth include impaction, delayed eruption, ectopic eruption, dental overcrowding, teeth spatial disorders, and the formation of follicular cysts. The treatment of supernumerary teeth depends on their type, position and possible complications detected in clinical and radiography examination. Extraction is advisable when incisor root development is complete in order to avoid any disorder in permanent dentition. To date, there is no clear consensus as to the best time to surgically extract an unerupted supernumerary tooth.
